# Identifying App-Based Meditation Habits and the Associated Mental Health Benefits: Longitudinal Observational Study

**DOI:** 10.2196/27282

**Published:** 2021-11-04

**Authors:** Chad Stecher, Vincent Berardi, Rylan Fowers, Jaclyn Christ, Yunro Chung, Jennifer Huberty

**Affiliations:** 1 College of Health Solutions Arizona State University Phoenix, AZ United States; 2 Department of Psychology Chapman University Orange, CA United States

**Keywords:** behavioral habits, habit formation, mindfulness meditation, mental health, mHealth, mobile health, dynamic time warping, mobile phone

## Abstract

**Background:**

Behavioral habits are often initiated by contextual cues that occur at approximately the same time each day; so, it may be possible to identify a reflexive habit based on the temporal similarity of repeated daily behavior. Mobile health tools provide the detailed, longitudinal data necessary for constructing such an indicator of reflexive habits, which can improve our understanding of habit formation and help design more effective mobile health interventions for promoting healthier habits.

**Objective:**

This study aims to use behavioral data from a commercial mindfulness meditation mobile phone app to construct an indicator of reflexive meditation habits based on temporal similarity and estimate the association between temporal similarity and meditation app users’ perceived health benefits.

**Methods:**

App-use data from June 2019 to June 2020 were analyzed for 2771 paying subscribers of a meditation mobile phone app, of whom 86.06% (2359/2771) were female, 72.61% (2012/2771) were college educated, 86.29% (2391/2771) were White, and 60.71% (1664/2771) were employed full-time. Participants volunteered to complete a survey assessing their perceived changes in physical and mental health from using the app. Receiver operating characteristic curve analysis was used to evaluate the ability of the temporal similarity measure to predict future behavior, and variable importance statistics from random forest models were used to corroborate these findings. Logistic regression was used to estimate the association between temporal similarity and self-reported physical and mental health benefits.

**Results:**

The temporal similarity of users’ daily app use before completing the survey, as measured by the dynamic time warping (DTW) distance between app use on consecutive days, significantly predicted app use at 28 days and at 6 months after the survey, even after controlling for users’ demographic and socioeconomic characteristics, total app sessions, duration of app use, and number of days with any app use. In addition, the temporal similarity measure significantly increased in the area under the receiver operating characteristic curve (AUC) for models predicting any future app use in 28 days (AUC=0.868 with DTW and 0.850 without DTW; *P*<.001) and for models predicting any app use in 6 months (AUC=0.821 with DTW and 0.802 without DTW; *P*<.001). Finally, a 1% increase in the temporal similarity of users’ daily meditation practice with the app over 6 weeks before the survey was associated with increased odds of reporting mental health improvements, with an odds ratio of 2.94 (95% CI 1.832-6.369).

**Conclusions:**

The temporal similarity of the meditation app use was a significant predictor of future behavior, which suggests that this measure can identify reflexive meditation habits. In addition, temporal similarity was associated with greater perceived mental health benefits, which demonstrates that additional mental health benefits may be derived from forming reflexive meditation habits.

## Introduction

### Background

Practicing healthier daily behaviors would improve many important physical and mental health outcomes for adults in the United States [1-5]. However, even when healthy behaviors are successfully initiated, many people find it difficult to maintain them as long-term habits [6-8] and thus do not attain the corresponding health benefits. One theory of habitual behaviors from psychology and neuroscience contends that habits are unconsciously or reflexively cued by environmental stimuli [9-11]. By repeatedly pairing an environmental or contextual cue with the performance of a desired behavior, our brains routinize the cue-behavior association, reducing the use of deliberative cognitive processes to instigate the daily performance of the behavior [12-14]. Recent research suggests that these contextually cued reflexive responses underlie many of our daily behaviors [9,15], and reflexively instigated habits are a commonly reported behavioral strategy among those who successfully maintain healthy habits, such as daily medication adherence and physical activity [16-20]. However, research has yet to examine mindfulness meditation practices, where the reduction in cognitive effort associated with reflexively instigating meditation [20-22] may enhance the mindfulness experience and increase the corresponding mental health benefits.

An additional limitation to the existing psychology research on habitual behaviors has been the reliance on self-reported measures of habit strength [23]. Habits are theorized to be unconsciously initiated, and as such, individuals should not be able to accurately recall their experience of performing a habitual behavior. Thus, self-reported measures are more likely to capture an individual’s perceived self-efficacy or fluency in their behavior [24]. The historical reliance on survey-based habit measures stemmed from a lack of detailed, longitudinal behavioral data necessary for observing daily behavioral patterns. With the recent advent and popularity of mobile health (mHealth) tools that collect precise, high-frequency data on users’ daily behaviors, there is a new opportunity for developing more objective indicators of daily habits. These data-driven measures offer the potential to more accurately describe the habit formation process and inform the design of new mHealth interventions that can more successfully promote the formation of healthier habits. As this type of high-frequency daily data are available for many health behaviors, such as physical activity (via wearable fitness trackers), medication adherence (via pill bottles with electronic caps), and mobile phone app–based mindfulness meditation, objective identifiers of reflexive habits will also allow the research on habitual behaviors to be translated across behavioral settings with a higher degree of fidelity.

### Goals of This Study

The 2 aims of this study are to construct and test an objective indicator of contextually cued mindfulness meditation habits and to estimate the association between this indicator and improvements in physical and mental health. For this research, we examined detailed observations of mindfulness meditation practices among users of *Calm,* a popular commercial meditation smartphone app. Our novel indicator of contextually cued habits was constructed to capture the temporal similarity of daily app use (ie, using the app at approximately the same time of the day) based on the dynamic time warping (DTW) distance between app use on consecutive days (detailed below). Existing research has shown that most contextually cued habits are performed at approximately the same time and in the same location each day [25,26], which motivates the use of temporal similarity to identify reflexively instigated meditation habits. However, as contextually cued habits are rarely based strictly on time, DTW is used to flexibly measure temporal changes in app-use patterns. For example, a person’s daily meditation habit could be cued by finishing lunch or arriving home after work, both of which may occur at slightly different times each day. Accordingly, we apply the DTW measure to capture the broad changes in daily app use start time, duration, or both, while allowing for small daily variations in these dimensions. As a large difference in temporal similarity between 2 days signals that the individual was meditating with the app in a different pattern on those days, we hypothesize that our measure of temporal similarity will indicate when the individual’s meditation behavior occurred outside of their usual behavioral context and routines. Admittedly, not all reflexive habits are performed at approximately the same time each day; however, our approach aims to identify most meditation habits that are initiated by temporally similar contextual cues.

In this study, we evaluate our measure of temporal similarity as an indicator of meditation habits by estimating the relative importance of temporal similarity for predicting users’ future app use. As contextually cued habits are known to underlie many of our long-term daily behaviors, we hypothesize that our temporal similarity measure will successfully identify many users’ reflexive meditation habits and thus significantly predict users’ future behavior. We test this hypothesis by comparing the predictive strength of temporal similarity with measures of the frequency and duration of app use [27-31]. Toward the second aim of this study, we then estimate the association between our temporal similarity measure and perceived physical and mental health benefits from using the meditation app. This second set of analyses was exploratory in nature and was designed to investigate the potential relationship between reflexive medication habits and changes in physical and mental health.

This is the first study to offer an objective indicator of reflexive meditation habits based on detailed mHealth data. This measure will allow researchers to better describe the habit formation process and to better target and measure future behavioral interventions for promoting healthier habits. This study is also the first to examine the potential mental health benefits of forming a mindfulness meditation habit, which provides new directions for future mHealth interventions that aim to improve mental health outcomes.

## Methods

### Recruitment and Data Description

We used longitudinal mindfulness meditation app user data from the commercial app Calm, which had >2 million paying subscribers at the time of data collection. Subscribers in December 2019 who were (1) aged at least 18 years, (2) had a subscription expiration that was at least 2 months away, and (3) had opened at least one email from Calm in the past 90 days were recruited via email to complete a survey on their perceived sleep quality improvements from using the app. These eligibility criteria were used to recruit persistent users of Calm who likely had high intrinsic motivation for meditation, which helps our analyses by reducing the potentially confounding influence of motivation on our ability to identify reflexive meditation habits; the average length of time since first subscribing to Calm was 11.5 months (SD 10.4 months) in this sample. The survey assessment also contained measures of users’ socioeconomic status, self-reported app use, physical and mental health status, and perceptions of the physical and mental benefits they experienced after using the app. The survey was approved by the institutional review board of the Arizona State University (STUDY00009725), and the results and additional details on the survey methods and findings have been published elsewhere [32].

Minute-level app use data for the same set of survey respondents were used to develop an objective identifier of habitual app use to predict future use of the app after completion of the survey and to estimate the association with perceived mental health benefits from using the app. The minute-level data set was compiled from all app sessions that were observed 6 months before (June 2019) to 6 months after (June 2020) respondents completed the survey in December 2019. The Calm app offers a wide range of mindfulness content, and a session was recorded when using any one of the following 7 session types: guided or timed meditations, sleep stores, breath training exercise (*breathe*), music, nature sounds (*soundscape*), in-depth audio classes (*masterclass*), and video lessons on mindful movement routines (*body*). For each observed session, the data contained information on the session’s start time, duration, type, title of the specific content, and whether the session was completed.

### Statistical Analysis

**Figure 1:**
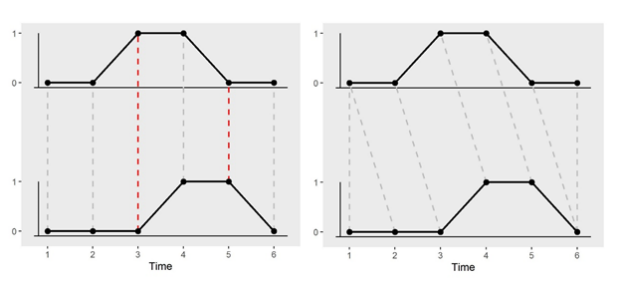
Visualization of the dynamic time warping (DTW) distance between 2 consecutive days of use, where 0 indicates not using the app and 1 indicates app use during the indicated period. The left panel shows the Euclidean distance between 2 days of equal total use (ie, two time periods with a 1) but the use pattern is shifted in time. As shown on the right panel, DTW compresses or stretches a time series so that the points on one day are mapped to nearby points in the other day that have similar values. The Euclidean distance between these 2 days is 2, whereas the DTW distance is 0. DTW: dynamic time warping.

To characterize app use before and after completing the survey, we calculated the number of total sessions used, number of unique sessions used, duration of use, start time, and whether any app use occurred on a given day [27-31]. In addition to these measures, temporal similarity was estimated by converting each day in our sample period into a 1440-minute time series of 0s (when the app was not in use) and 1s (when the app was being used) and then calculating the DTW distance between the time series of app use on consecutive days. As shown in Figure 1, the DTW distance measure is a more flexible calculation than the Euclidean distance between any two 1440-minute time series as it compares the use on one day to use over a wider range of minutes on the other day. Although the Euclidean distance compares use between the exact same minute on each day, Figure 1 shows that the DTW distance is calculated by comparing use on one day with a similar pattern of use that occurred slightly later on the next day. In this way, DTW distance does not penalize users for engaging in the same overall pattern of daily app use that is simply shifted a few minutes earlier or later on a given day. This measure of temporal similarity was calculated using the *dtw* package in R version 4.0.1 (R Foundation for Statistical Computing) and additionally modified to penalize nonuse on consecutive days (see Multimedia Appendix 1 for details). In this way, the final measure of temporal similarity can be interpreted as the average percentage of daily minutes that are inconsistently performed between consecutive days. See Multimedia Appendix 2 for a visualization of the DTW calculation and other attributes found in the Calm app use data.

Each of the objective measures of app use, including frequency, duration, and the temporal similarity measure, were calculated daily for each user and averaged within nonoverlapping 14-day intervals. We then analyzed changes in these measures over multiple intervals to describe how app use patterns changed over time. As some of the users had infrequent app use at the start of our sample period, each user was assigned a unique start date based on the first Monday after at least 5 days of use within a 14-day period. We started each user on a Monday to control for the observed differences in behavior between weekdays and weekends. The objective measures of app use were then defined for all users over 10 nonoverlapping 14-day intervals preceding the date of survey completion. The use of 14-day intervals was chosen so that each interval would contain an equal number of weekdays and weekends, and the results were largely unchanged when these intervals were shortened or widened. For users without 10 nonoverlapping 14-day intervals between their start date and the date of survey completion, additional 14-day intervals were constructed after the survey date so that all users had 10 intervals (140 days) over which the aforementioned objective measures were calculated. These additional postsurvey intervals were not used to analyze the survey-based mental health outcomes (second aim) but were used to test the ability of temporal similarity to predict future app use (first aim).

Finally, these objective measures of app use were calculated based on all observed sessions, as well as separately by session type and session timing, stratified by *Morning* (4 AM to 12 PM), *Evening* (12 PM to 8 PM), or *Night* (8 PM to 4 AM the next day). These measures, along with users’ socioeconomic status, were summarized using means and SDs for continuous variables and counts and percentages for dichotomous variables; group differences in dichotomous variables were compared using the *prtest* command in Stata/MP 16.1 (StataCorp) [33].

Toward our first aim of testing temporal similarity as an indicator of reflexive meditation habits, we estimated the predictive ability of temporal similarity on 3 dichotomous measures of future app use: any app use 28 days later, any app use 6 months later, and an identifier for whether the total duration of app use during the 28-day period following the survey was above the median duration during this period. For each of these 3 outcomes, 3 predictive modeling techniques were used. First, logistic regression was used to estimate the associations between each dichotomous outcome and all available objective app-use measures. Second, the same logistic models with and without the DTW-based temporal similarity measures were used to calculate the receiver operating characteristic (ROC) curves and the corresponding areas under the ROC curve (AUCs), a measure of model fit. AUCs from the 2 models (ie, with and without the DTW-based temporal similarity measure) were compared using a chi-square test [34] performed by the *roccomp* command in Stata/MP 16.1. Finally, using the same set of objective app-use measures, we estimated the relative importance of each app-use measure using a random forest algorithm from the *sklearn.ensemble.RandomForestClassier* command in Python 3.8.3 (Python Software Foundation) [35].

To investigate the relationship between reflexive meditation habits and changes in health, we used logistic regression to estimate the association between perceived physical and mental benefits from using the app and the objective app-use measures, including temporal similarity. Physical and mental benefits were measured from survey questions that separately asked whether 7 different physical health conditions (hypertension, high cholesterol, asthma, emphysema, other lung diseases, heart disease, cancer, pain, and arthritis) and 3 different mental health conditions (anxiety, posttraumatic stress disorder, and depression) were *improved* and whether these measures were *very improved*. The dichotomous measure of whether physical health was *improved* was set equal to 1 if participants responded with an affirmative response (ie, yes, the condition was improved) to any of the physical health conditions and equal to 0 otherwise. Similar procedures were used to construct the dichotomous measure of *improved* mental health and to construct separate dichotomous measures for physical and mental health reported as *very improved*. Logistic regressions estimated the association between these dichotomous measures of physical and mental health improvements and objective app-use measures, controlling for users’ socioeconomic status. To better compare model estimates for the different objective app-use measures, a logarithm transformation was applied to each app-use measure so that each association measured the correlation between a 1% increase in the indicated behavioral measure and the odds of experiencing improved health from using the app. Similar log transformations were also applied to the predictive logistic models described above to further improve comparability across the estimated statistical relationships. The logistic regressions were performed by the *logit* command in Stata/MP 16.1

### Data Exclusion

Survey respondents that did not have sufficient app use to meet the start date definition detailed above were not included in these analyses (n=2239). However, their irregular app use provides strong evidence that these users did not habitually use the app. Therefore, the results of this study and subsequent discussion serve to describe how *regular* users of the app may benefit from temporally consistent meditation habits, both in their odds of persistent app use and experiencing health benefits.

## Results

### Sample Characteristics

The sample of Calm users was aged between 21 and 87 years (mean 48.0 years, SD 14.2 years), was primarily female (2359/2771, 86.06%) and White (2391/2771, 86.29%), with a median household income of US $80,000 (mean US $105,927, SD US $86,940.20). Table 1 illustrates the demographic and socioeconomic characteristics, which are largely the same between those with above and equal to or below the median number of meditation sessions used during the 14 days before completing the survey (median=2 sessions). Between both groups, less than two-thirds of users worked full-time. Approximately 43.27% (1199/2771) reported having at least one mental health condition (stress, depression, or anxiety), and approximately 39.01% (1081/2771) reported at least one chronic physical health condition (eg, emphysema or cancer). Household income is presented as a categorical variable in Table 1 to better describe the distribution of income in the sample, which was >US $61,000 for 61.1% (1693/2771) of the participants.

**Table 1:** Sample demographics by observed use of meditation sessions (N=2771).

Characteristics		Total sample, n (%)	Above median meditation sessions^a^ (n=1546), n (%)	Equal or below median meditation sessions^a^ (n=1225), n (%)	Difference (percentage points)	*P* value^b^
**Age (years)**						
	18-30	325 (12.23)	186 (12.03)	139 (11.35)	−0.74	.56
	31-40	581 (20.97)	317 (20.5)	264 (21.55)	1.05	.50
	41-50	607 (21.91)	337 (21.8)	270 (22.04)	0.24	.88
	51-60	549 (19.81)	306 (19.79)	243 (19.84)	0.04	.98
	61-70	443 (15.99)	254 (16.43)	189 (15.43)	−1.00	.48
	71-80	141 (5.09)	73 (4.72)	68 (5.55)	0.83	.32
	>81	123 (4.44)	72 (4.66)	51 (4.16)	−0.49	.53
**Race**						
	White	2391 (86.29)	1312 (84.86)	1079 (88.08)	3.22	.01
	Asian	78 (2.81)	51 (3.3)	27 (2.2)	−1.09	.08
	Native American	6 (0.22)	3 (0.19)	3 (0.24)	0.05	.78
	Black	73 (2.63)	45 (2.91)	28 (2.29)	−0.63	.31
	Biracial	80 (2.89)	52 (3.36)	28 (2.29)	−1.08	.09
	Race other	222 (8.01)	131 (8.47)	91 (7.43)	−1.04	.31
	Hispanic	165 (6.05)	98 (6.34)	67 (5.47)	−0.94	.31
Sex (female)		2359 (86.06)	1270 (83.15)	1089 (88.9)	6.66	<.001
**Household income (US $)**						
	<21,000	127 (4.58)	77 (4.98)	50 (4.08)	−1.42	.21
	21,000-60,000	469 (16.92)	242 (15.65)	227 (18.53)	3.55	.07
	61,000-100,000	521 (18.8)	304 (19.66)	217 (17.71)	−3.34	.10
	>100,000	671 (24.21)	368 (23.8)	303 (24.73)	0.57	.79
Employed full-time		1664 (60.71)	910 (59.63)	754 (61.55)	2.42	.20
**Education**						
	Bachelor’s degree	1030 (37.17)	570 (36.87)	460 (37.55)	0.65	.73
	Graduate degree	982 (35.44)	559 (36.16)	423 (34.53)	−1.67	.36
**Health status**						
	Mental health condition	1199 (43.27)	680 (43.98)	519 (42.37)	−1.62	.39
	Physical health condition	1081 (39.01)	592 (38.29)	489 (39.92)	1.63	.38
	Only mental health diagnosis	618 (22.3)	368 (23.8)	250 (20.41)	−3.40	.03

^a^Number of meditation sessions measured over the 14 days before survey completion; median number of meditation sessions over the 14 days before the survey was 2 sessions.

^b^*P* values from proportion tests comparing those with above and those with equal to or below the median number of meditation sessions during the 2 weeks before survey completion.

Most users, approximately 69.87% (1936/2771), reported using the app ≥5 times per week, as shown in Table 2. Importantly, 38% (1053/2771) of users noticed improved mental health status from using the app, whereas 19.38% (537/2771) experienced improved physical health. Table 2 also illustrates the mental health benefits from mindfulness meditation, as the users with above the median number of meditation sessions during the 14 days before completing the survey were 3.5 percentage points (*P*=.05) more likely to experience improved mental health and 8.5 percentage points (*P*<.001) more likely to experience very improved mental health than those with equal to or below the median number of meditation sessions.

**Table 2:** Self-reported use and health benefits by observed meditation sessions (N=2771).

Characteristics			Total sample, n (%)		Above median meditation sessions^a^ (n=1546), n (%)		Equal or below median meditation sessions^a^ (n=1225), n (%)		*P* value^b^
**Self-reported app use**									
	1-2 days/week	190 (6.86)		110 (7.12)		80 (6.53)		.55	
	3-4 days/week	549 (19.81)		280 (18.11)		269 (21.96)		.01	
	5-7 days/week	1939 (69.97)		1099 (71.09)		840 (68.57)		.15	
	Use any meditation features	1610 (58.1)		1119 (72.38)		491 (40.08)		<.001	
	Use any sleep stories features	2282 (82.35)		1165 (75.36)		1117 (91.18)		<.001	
	Use in mornings	837 (30.21)		689 (44.57)		148 (12.08)		<.001	
	Use in evenings	809 (29.2)		513 (33.18)		296 (24.16)		<.001	
	Use at night	2561 (92.42)		1376 (89)		1185 (96.73)		<.001	
	Try to consistently use at night	1575 (56.84)		790 (51.1)		785 (64.08)		<.001	
**Perceived health benefits**									
	Improved mental health	1053 (38)		612 (39.59)		441 (36)		.05	
	Improved physical health	537 (19.38)		327 (21.15)		210 (17.14)		.008	
	Improved mental health (only)	771 (27.82)		438 (28.33)		333 (27.18)		.50	
	Very much improved mental health	482 (17.39)		327 (21.15)		155 (12.65)		<.001	
	Very much improved physical health	119 (4.29)		75 (4.85)		44 (3.59)		.10	
	Very much improved mental health (only)	430 (15.52)		295 (19.08)		135 (11.02)		<.001	

^a^Number of meditation sessions measured over the 14 days before survey completion; median number of meditation sessions over the 14 days before the survey was 2 sessions.

^b^*P* values from proportion tests comparing those with above and those with equal to or below the median number of meditation sessions during the 2 weeks before completing the survey.

Table 3 presents the mean, SD, and maximum values of the objective measures of app use calculated over the 14 days before survey completion. On average, the likelihood of using any session type on a given day was 55.7%, the likelihood of using meditation features was 28.4%, and the likelihood of using sleep stores was 32% over this period. Approximately 1.14 (SD 2.26) sessions of any type were used per day, with 0.35 (SD 1.11) sessions occurring in the mornings and 0.66 (SD 1.21) sessions occurring in the evenings. Meditation sessions were performed with equal frequency in the mornings—approximately 0.17 (SD 0.36) sessions per day—and the evenings—approximately 0.16 (SD 0.34) sessions per day—whereas sleep stories were largely performed at night (mean 0.37 sessions per day, SD 0.51 sessions per day). A similar pattern was observed for users’ average duration in minutes of app use per day by session type, where meditation sessions were used for approximately 2.30 (SD 5.91) minutes in the mornings and 2.84 (SD 6.56) minutes in the evenings, and sleep stories were used for an average of 11.54 (SD 16.19) minutes in the evenings. Finally, the average DTW distance measure across all session types, which incorporated the penalty for nonuse and was standardized by users’ average duration of daily use over these 14 days, was equal to 0.539 (SD 0.35). In contrast, the DTW distance measured just among meditation and sleep story features was smaller, at approximately 0.489 (SD 0.44) and 0.474 (SD 0.45), respectively.

**Table 3:** Objective app use measures over 14 days before survey completion (N=2771).

Characteristics	Value, mean (SD)	Maximum
Any session/day	0.557 (0.37)	1.000
Meditation sessions/day	0.284 (0.36)	1.000
Sleep stories sessions/day	0.320 (0.35)	1.000
Sessions/day in mornings	0.351 (1.11)	48.286
Sessions/day in evenings	0.121 (0.32)	6.786
Sessions/day at night	0.666 (1.21)	45.643
Sessions/day weekdays	0.836 (1.87)	81.643
Sessions/day weekends	0.302 (0.46)	12.286
Sessions/day total	1.139 (2.26)	93.929
Meditation sessions/day in mornings	0.173 (0.36)	4.357
Meditation sessions/day in evenings	0.067 (0.19)	2.571
Meditation sessions/day at night	0.168 (0.34)	3.214
Sleep stories sessions/day in mornings	0.081 (0.25)	2.786
Sleep stories sessions/day in evenings	0.025 (0.12)	2.357
Sleep stories sessions/day at night	0.369 (0.51)	4.929
Duration/day in mornings (minutes)	7.149 (16.82)	352.309
Duration/day in evenings (minutes)	2.137 (5.86)	81.578
Duration/day at night (minutes)	18.043 (25.83)	596.222
Duration/day weekdays (minutes)	19.737 (29.93)	768.200
Duration/day weekends (minutes)	7.592 (11.97)	239.941
Meditation duration/day in mornings (minutes)	2.295 (5.91)	142.167
Meditation duration/day in evenings (minutes)	0.814 (2.36)	35.801
Meditation duration/day at night (minutes)	2.836 (6.56)	83.739
Sleep stories duration/day in mornings (minutes)	2.480 (7.62)	91.848
Sleep stories duration/day in evenings (minutes)	0.750 (3.51)	78.647
Sleep stories duration/day at night (minutes)	11.535 (16.19)	169.736
DTW^a^ distance	0.539 (0.35)	3.207
DTW distance (only meditation sessions)	0.489 (0.44)	1.386
DTW distance (only sleep stories)	0.474 (0.45)	1.902

^a^DTW: dynamic time warping.

### Predictive Models

The first set of analyses used the objective measure of app use over 140 days to predict users’ future app use to test the ability of temporal similarity to identify reflexive habits. Table 4 displays the exponentiated logistic regression coefficients and 95% CIs using the objective app-use measures to predict the 3 dichotomous measures of future app use. The table displays the results for all objective app-use measures calculated over the 6th through the 10th 14-day interval after each user’s start date. The app-use measures calculated over intervals 1-5, and the demographic controls displayed in Table 1 were also included in each logistic regression model but were suppressed from Table 4 for ease of presentation.

**Table 4:** Objective measures in the 10th (2-week) interval predicting future app use (N=2771).^a^

Predictor		Odds ratio (95% CI)					
		Any use 28 days later	Any use 28 days later	Any use 6 months later	Any use 6 months later	High duration in next 28 days	High duration in next 28 days
**Days of any use**							
	Interval 10	3.378^b^ (2.32-4.92)	4.282^b^ (2.65-6.93)	1.527^c^ (1.11-2.10)	1.397 (0.91-2.15)	1.148 (0.66-1.31)	1.676 (0.38-1.80)
	Interval 9	1.955^b^ (1.37-2.79)	2.159^c^ (1.35-3.45)	1.278 (0.91-1.79)	1.197 (0.78-1.84)	1.187 (0.56-1.20)	1.606 (0.32-1.85)
	Interval 8	1.119 (0.72-1.74)	1.076 (0.64-1.82)	0.851 (0.56-1.30)	1.089 (0.66-1.81)	1.252 (0.82-1.91)	1.471^d^ (1.13-1.97)
	Interval 7	0.856 (0.54-1.35)	1.127 (0.65-1.95)	1.165 (0.76-1.78)	1.346 (0.80-2.26)	1.106 (0.72-1.70)	1.195 (0.54-1.71)
	Interval 6	1.054 (0.67-1.67)	1.202 (0.71-2.04)	1.115 (0.74-1.68)	1.171 (0.71-1.95)	1.065 (0.71-1.59)	1.203 (0.64-1.63)
**Total sessions**							
	Interval 10	1.347 (0.72-2.52)	1.561 (0.82-2.97)	1.107 (0.58-2.11)	1.024 (0.54-1.95)	1.140 (0.64-2.02)	0.785 (0.37-1.67)
	Interval 9	1.508 (0.83-2.73)	1.484 (0.83-2.66)	0.701 (0.39-1.27)	0.757 (0.41-1.41)	0.887 (0.50-1.57)	1.566 (0.78-3.14)
	Interval 8	0.899 (0.52-1.55)	0.926 (0.53-1.61)	1.100 (0.62-1.94)	1.019 (0.57-1.82)	1.322 (0.74-2.35)	1.334 (0.61-2.93)
	Interval 7	1.127 (0.61-2.07)	1.072 (0.58-1.99)	0.803 (0.45-1.42)	0.835 (0.46-1.53)	1.149 (0.68-1.95)	1.564 (0.74-3.32)
	Interval 6	0.809 (0.46-1.41)	0.839 (0.48-1.46)	1.842^d^ (1.02-3.34)	1.831 (0.99-3.40)	1.003 (0.56-1.79)	1.043 (0.46-2.34)
**Total duration**							
	Interval 10	0.953 (0.86-1.06)	0.857^d^ (0.75-0.97)	1.022 (0.94-1.11)	1.012 (0.92-1.11)	1.108^d^ (1.02-1.20)	1.272^c^ (1.07-1.51)
	Interval 9	0.837^c^ (0.74-0.95)	0.796^b^ (0.70-0.90)	1.005 (0.92-1.10)	0.984 (0.89-1.08)	0.980 (0.89-1.08)	1.193^d^ (1.00-1.42)
	Interval 8	1.045 (0.90-1.22)	1.004 (0.86-1.17)	1.020 (0.91-1.15)	1.016 (0.90-1.15)	0.889^d^ (0.79-1.00)	0.964 (0.80-1.16)
	Interval 7	1.024 (0.89-1.18)	1.022 (0.88-1.18)	1.055 (0.94-1.19)	1.055 (0.93-1.20)	0.912 (0.81-1.02)	1.011 (0.84-1.22)
	Interval 6	1.043 (0.90-1.20)	1.064 (0.92-1.23)	0.963 (0.85-1.08)	0.932 (0.82-1.06)	0.964 (0.86-1.08)	1.015 (0.83-1.25)
**DTW^e^ distance**							
	Interval 10	—^f^	0.716^d^ (0.68-0.92)	—	0.719^c^ (0.69-0.88)	—	0.401^c^ (0.12-0.81)
	Interval 9	—	0.924 (0.32-2.68)	—	0.689 (0.27-1.73)	—	0.896 (0.32-4.30)
	Interval 8	—	0.847^c^ (0.71-0.93)	—	0.695^d^ (0.51-0.81)	—	0.0642^c^ (0.01-0.29)
	Interval 7	—	0.972 (0.88-6.93)	—	0.941 (0.87-1.17)	—	0.178^d^ (0.04-0.77)
	Interval 6	—	0.842^d^ (0.71-0.92)	—	0.780 (0.30-2.00)	—	0.200 (0.04-1.02)
All-use-measure intervals 5-1		✓^g^	✓	✓	✓	✓	✓
Demographic and SE controls		✓	✓	✓	✓	✓	✓

^a^This table displays the odds ratios (exponentiated coefficients) from separate logistic regression models of each outcome indicated by the column headers on the objective app-use measures indicated by the row labels; 95% CIs are displayed in parentheses. Each objective app-use measure was log transformed to improve the comparability of the estimated relationships, and all models, in addition, included measures of users’ demographic and socioeconomic characteristics and the objective app-use measures estimated over intervals 1-5.

^b^*P*<.05.

^c^*P*<.01.

^d^*P*<.001.

^e^DTW: dynamic time warping.

^f^The dynamic time warping distance variables were excluded from one model of each outcome and included in the second model for the same outcome to compare the variable importance results from these 2 approaches.

^g^The indicated variables were also assessed in the model.

Table 4 shows that the number of days of any app use in the 10th 14-day interval (closest interval to the outcomes) was the strongest predictor of the odds of any future use 28 days later (odds ratio [OR] 3.378, 95% CI 2.32-4.92) and the odds of any future use 6 months later (OR 1.527, 95% CI 1.11-2.10). However, this measure was not significantly associated with the odds of having a high duration of total app use over the following 28 days. The results also demonstrated that the total number of sessions was a weak predictor of future app use across all 3 outcomes. In addition, total duration was a significant predictor of the odds of having a high duration over the following 28 days (OR 1.108, 95% CI 1.02-1.20). Importantly, the DTW measure of temporal similarity was significantly associated with all 3 outcomes describing future behavior. Specifically, a 1% increase in DTW distance in the 10th 14-day interval (ie, less similar timing of daily app use), which corresponds to a 0.015 SD increase from the mean DTW distance, is associated with an OR of 0.719 (95% CI 0.69-0.88) of any app use 6 months later.

**Figure 2:**
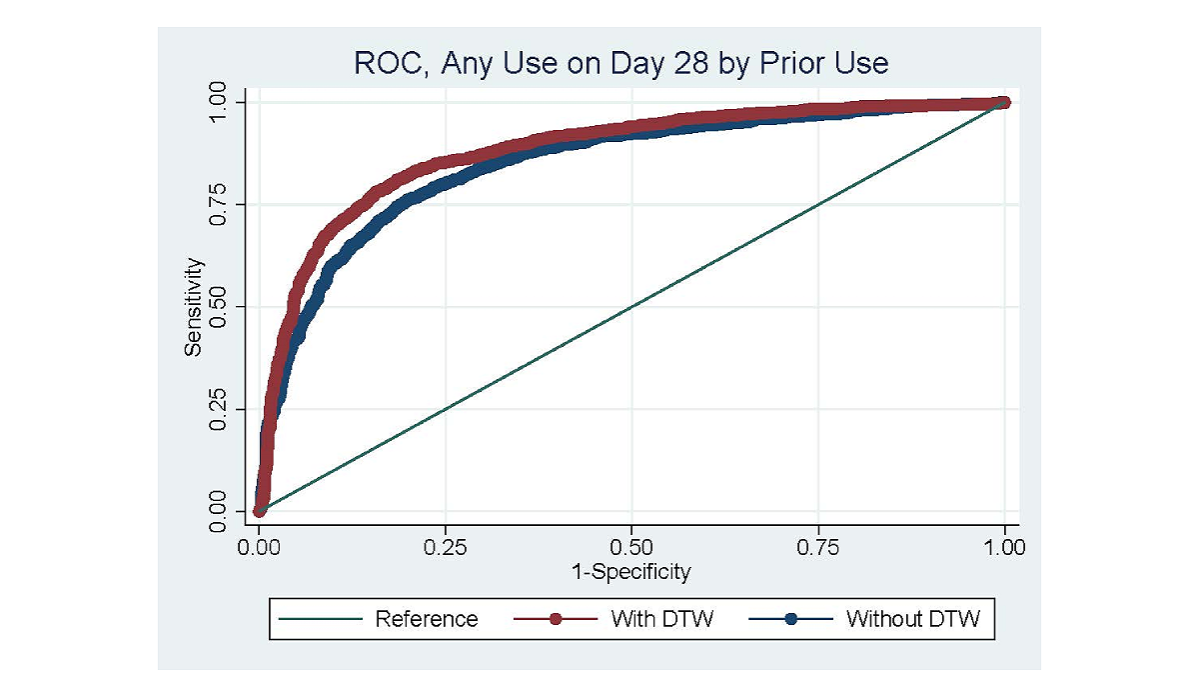
The receiver operating characteristic (ROC) curves for logistic regression models that include objective app-use measures calculated over the first 10 14-day intervals from users’ start date and demographic controls for predicting the likelihood of any future app use 28 days later. The 2 curves show the models without and with the dynamic time warping (DTW) distance measures, and the area under the ROC curves are equal to 0.850 and 0.868 for the models without and with DTW distance measures, respectively. DTW: dynamic time warping; ROC: receiver operating characteristic.

The first panel of Table 5 presents a second statistical test of the predictive value of the temporal similarity measure by comparing the AUC from the same set of logistic regression models shown in Table 5. The AUC without DTW-based temporal similarity measures was 0.818 when predicting any future use 28 days later, and this area was increased by 0.003 (*P=*.02) when the DTW distance measures were included in the model. To visualize this improvement in prediction accuracy, these 2 ROC curves are displayed in Figure 2. The second panel of Table 5 performs the same comparison between logistic regression models with and without the DTW distance measures, where all objective app-use measures were separately calculated over only the weekdays and weekends in each 14-day interval. These more granularly defined measures of app use improved the overall model fit, as the AUC increased to 0.820 when predicting the odds of any use 28 days later without DTW measures. The AUC increased to 0.828 (*P*<.001) when including the DTW measures defined over weekdays and weekends. Finally, the third panel in Table 5 displays the results when all objective app-use measures were separately calculated for each of the 7 session types and between weekdays and weekends. When the DTW distance measures were included in these logistic regression models, the AUC was significantly increased for the prediction of any future use 28 days later (*P*<.001), any use 6 months later (*P*<.001), and having a high duration of total app use over the next 28 days (*P*<.001).

**Table 5:** Area under the receiver operating characteristics curve (AUC) with and without dynamic time warping (DTW) to predict future app use.

Predictor			AUC				
			Any use 28 days later		Any use 6 months later		Above median duration of use over next 28 days
**By aggregating all sessions**							
	Not including DTW	0.818		0.729		0.953	
	Including DTW	0.821		0.732		0.953	
	Difference	0.003		0.003		0.000	
	*P* value	0.02		0.10		.77	
**By session timing (weekday, weekend)**							
	Not including DTW	0.820		0.741		0.950	
	Including DTW	0.828		0.747		0.956	
	Difference	0.008		0.008		0.006	
	*P* value	<.001		.006		<.001	
**By session type and timing**							
	Not including DTW	0.850		0.802		0.958	
	Including DTW	0.868		0.821		0.963	
	Difference	0.018		0.019		0.005	
	*P* value	<.001		<.001		<.001	

A second demonstration of the importance of temporal similarity for predicting future app use is presented in Multimedia Appendix 3, which displays the variable importance statistics from random forest models that used the objective app-use measures defined over all session types and timing to predict the same 3 dichotomous measures of future app use presented in Tables 4 and 5. The results show that at least one of the DTW distance measures is among the top 5 predictors of all 3 outcomes, further reinforcing the important association between temporal similarity and behavioral persistence in this setting.

### Associated Physical and Mental Benefits

The second set of analyses estimated the association between the objective app-use measures and self-reported mental and physical benefits from using the app. Table 6 displays the exponentiated coefficients from logistic regression models of the dichotomous outcomes indicating users’ self-reported *improved* or *very improved* mental or physical health from using the app on the demographic and socioeconomic dimensions presented in Table 1. From Table 6, we can see that older adults were significantly less likely to have reported experiencing mental health improvements but were more likely to have experienced physical health improvements. Specifically, adults aged between 71 and 80 years had an OR 0.239 (95% CI 0.15-0.39) of reporting improved mental health and an OR 2.35 (95% CI 1.15-4.80) of reporting improved physical health relative to users aged under 31 years. These estimated associations highlight the need to include user demographics in subsequent models for predicting perceived health benefits.

**Table 6:** Self-reported health benefits on user demographics (N=2771).

Demographics			Odds ratio (95% CI)^a^						
			Improved mental health		Improved physical health		Very much improved mental health		Very much improved physical health
**Age (years)**									
	<31	Reference		Reference		Reference		Reference	
	31-40	0.861 (0.649-1.143)		1.388 (0.817-2.359)		0.815 (0.578-1.150)		2.022 (0.635-6.442)	
	41-50	0.610^b^ (0.458-0.813)		3.586^b^ (2.190-5.871)		0.763 (0.539-1.081)		3.409^c^ (1.122-10.36)	
	51-60	0.385^b^ (0.286-0.519)		4.729^b^ (2.907-7.694)		0.562^d^ (0.391-0.808)		6.412^b^ (2.236-18.38)	
	61-70	0.383^b^ (0.279-0.526)		6.626^b^ (4.040-10.87)		0.613^c^ (0.418-0.899)		10.24^b^ (3.583-29.29)	
	71-80	0.239^b^ (0.148-0.388)		8.232^b^ (4.588-14.77)		0.366^d^ (0.191-0.700)		9.842^b^ (2.911-33.27)	
	>81	0.449^d^ (0.274-0.737)		2.348^c^ (1.150-4.795)		0.712 (0.391-1.296)		3.445 (0.746-15.92)	
**Sex**									
	Male	Reference		Reference		Reference		Reference	
	Female	1.608^b^ (1.256-2.060)		0.963 (0.715-1.296)		1.318 (0.965-1.801)		1.490 (0.776-2.861)	
**Race**									
	Other	Reference		Reference		Reference		Reference	
	White	0.936 (0.679-1.291)		0.785 (0.545-1.131)		0.873 (0.601-1.268)		0.879 (0.470-1.644)	
	Asian	0.336^b^ (0.176-0.643)		0.818 (0.377-1.774)		0.397^c^ (0.165-0.956)		3.019^c^ (1.036-8.803)	
	Black	0.634 (0.351-1.145)		1.318 (0.706-2.458)		0.737 (0.355-1.531)		2.985^c^ (1.189-7.496)	
	Hispanic	0.731 (0.507-1.055)		1.048 (0.663-1.657)		1.273 (0.848-1.912)		2.801^d^ (1.431-5.483)	
Log (income)			0.918 (0.829-1.017)		0.888^c^ (0.789-0.999)		0.895^c^ (0.803-0.998)		0.836^c^ (0.707-0.989)
**Employment**									
	Not employed full-time	Reference		Reference		Reference		Reference	
	Employed full-time	0.847 (0.704-1.019)		0.857 (0.681-1.080)		0.929 (0.737-1.171)		0.933 (0.600-1.451)	
**Education**									
	Less than a college degree	Reference		Reference		Reference		Reference	
	Bachelor’s degree	0.787^c^ (0.642-0.966)		1.032 (0.800-1.333)		0.736^c^ (0.573-0.946)		0.918 (0.580-1.451)	
	Graduate degree	0.607^b^ (0.491-0.751)		0.860 (0.663-1.116)		0.583^b^ (0.447-0.760)		0.605 (0.365-1.003)	

^a^This table displays the odds ratios (exponentiated coefficients) from separate logistic regression models of each outcome indicated by the column headers on the demographic and socioeconomic characteristics indicated by the row labels.

^b^*P*<.001.

^c^*P*<.05.

^d^*P*<.01.

Table 7 shows the estimated association between improved mental and physical health and the objective measures of app use, conditional on the user demographic and socioeconomic dimensions presented in Table 6. The first panel (model 1) of Table 7 presents the exponentiated coefficients for a model containing the objective app-use measures averaged over the 6 weeks (3×14-day intervals) before survey completion. Conditional on the total number of sessions, total duration of use, and the total number of days with any use, a 1% increase in DTW distance over the 6 weeks before survey completion was associated with an OR 0.34 (95% CI 0.16-0.55) of experiencing improved mental health. The model also estimated a negative relationship between DTW distance and improved physical health; however, this relationship was not statistically significant.

**Table 7:** Self-reported health benefit on objective app use averaged over the past 6 weeks (N=2771).^a^

Characteristics			Value, odds ratio (95% CI)						
			Improved mental health		Improved physical health		Very much improved mental health		Very much improved physical health
**Model 1**									
	Total number of sessions	1.094 (0.822-1.456)		1.340 (0.965-1.862)		1.718^b^ (1.249-2.362)		1.879^c^ (1.131-3.121)	
	Total duration	0.955 (0.874-1.044)		0.929 (0.824-1.048)		0.822^b^ (0.733-0.921)		1.006 (0.826-1.225)	
	Total days with any use	1.244 (0.957-1.618)		1.233 (0.879-1.729)		1.836^b^ (1.316-2.562)		0.925 (0.535-1.601)	
	DTW^d^ distance	0.340^e^ (0.157-0.546)		0.687 (0.212-1.123)		0.231^e^ (0.026-0.487)		0.512^c^ (0.315-0.727)	
**Model 2**									
	DTW distance; meditation only	0.722^c^ (0.495-0.971)		0.601 (0.342-1.022)		0.436^b^ (0.277-0.688)		0.475 (0.201- 1.124)	
	Number of meditation sessions	1.660 (0.876-3.147)		2.180^c^ (1.051-4.522)		2.721^e^ (1.325-5.587)		0.410 (0.122-1.383)	
	Duration of meditation sessions	0.985 (0.927-1.048)		0.958 (0.888-1.033)		1.009 (0.937-1.087)		1.090 (0.956-1.242)	
	DTW distance; sleep stories only	0.715 (0.440-1.430)		0.868 (0.575-1.364)		0.567 (0.311-1.060)		0.914 (0.321-2.601)	
	Number of sleep story sessions	0.860 (0.541-1.366)		1.369^c^ (1.196-2.354)		1.184^c^ (1.059-2.026)		1.331^e^ (1.189-3.624)	
	Duration of sleep stories	1.042 (0.993-1.093)		1.021 (0.962-1.085)		1.044 (0.982-1.111)		1.023 (0.910-1.150)	
Demographic controls			✓^f^		✓		✓		✓

^a^This table displays the odds ratios (exponentiated coefficients) from separate logistic regression models of each outcome indicated by the column headers on the objective app-use measures indicated by the row labels for each of the 2 predictive models. Each objective app-use measure was log transformed to improve the comparability of the estimated relationships, and all models, in addition, included measures of users’ demographic and socioeconomic characteristics.

^b^*P*<.001.

^c^*P*<.05.

^d^DTW: dynamic time warping.

^e^*P*<.01.

^f^Demographic controls were also assessed in the model.

The second panel (model 2) in Table 7 presents the same set of objective app-use measures calculated for the 2 most commonly used session types: meditation and sleep stories. The results show that DTW distance for meditation sessions is a significant predictor of both improved and very improved mental health. Specifically, a 1% increase in DTW for meditation sessions was associated with an OR 0.72 (95% CI 0.50-0.97) of experiencing improved mental health and an OR 0.44 (95% CI 0.27-0.69) of experiencing very improved mental health from using the app. Changes in DTW distance for sleep story sessions were also negatively associated with the odds of experiencing improved (OR 0.72, 95% CI 0.44-1.43) and very improved mental health (OR 0.57, 95% CI 0.31-1.06); however, these relationships were not statistically significant nor were they significant for either measure of physical health improvement. In addition, the DTW distance measure was also a significant predictor of using the app for a greater number of meditation sessions and sleep stories over the next 28 days (Multimedia Appendix 4 and Table S1 of Multimedia Appendix 5).

## Discussion

### Principal Findings

The 2 aims of this study were to construct and test an objective indicator of reflexive meditation habits and to explore the association between meditation habits and mental health. Toward this first aim, we constructed and provided evidence that our novel measure of temporally similar app use strongly predicted future app use and, thus, is likely to indicate the presence of reflexive meditation habits. Specifically, after controlling for user demographics and common app use metrics, such as frequency and duration of app sessions, the temporal similarity of app use on consecutive days was shown to significantly predict the odds of any future app use and the duration of future use. That is, using the meditation app at roughly the same time each day was associated with greater persistence in meditation app use, which suggests that this measure of temporal similarity was able to indicate the formation of reflexive meditation habits.

We then found that there was a significant association between increased temporal similarity in daily meditation app use and improved mental health from using the app. This finding suggests that the reduced cognitive effort required to instigate meditation that results in a reflexive meditation habit may enable individuals to use more cognitive resources in their mindfulness meditation practice and, thus, experience greater mental health benefits. This interpretation is supported by research describing the impact of attentional resources [36] and mental effort [37-39] on the success of different meditation practices. As the goal of mindfulness meditation is to direct one’s attention to the present moment, these findings indicate that this practice may be easier to perform if meditation is instigated reflexively and without effortful deliberation. However, our study did not investigate the specific mechanism or mechanisms underlying the association between temporal similarity and improved mental health, which is an important area for future research.

### Additional Findings

Our descriptive results found that many observable demographic and socioeconomic characteristics were significantly associated with self-reported mental and physical health improvements. Conversely, only the individual’s age was significantly associated with measures of the likelihood and duration of future app use (Multimedia Appendix 3). These findings suggest that behavioral habits are equally experienced across demographic characteristics; however, future research assessing the health benefits of mindfulness meditation practices should carefully control for these significant demographic and socioeconomic factors. In addition, the results showed that larger improvements in mental health were reported among individuals who used more than the median number of meditation sessions in the 14 days before the survey, which adds additional evidence for the mental health benefits of mindfulness meditation.

### Limitations

Overall, this was a very active sample of app users, with a 55.7% average likelihood of using any app session on a given day over the study period. This is not surprising as the sample consisted of paying subscribers who responded to emails from the app and volunteered to take part in a study to help improve the app; so, the results of this research should be extrapolated to other user types with caution. Although the demographic and socioeconomic minorities of this sample did not display significantly different app-use patterns, the small sample size of these groups limits the statistical power of these comparisons. Future research on mindfulness meditation behaviors should aim to collect data from a wider demographic range of users to better characterize meditation habits and the mental health benefits experienced by all users.

Another limitation was the nature of the data set compiled for these analyses. Specifically, the objective measures of daily app use were compiled for the period around the date when users completed the survey on their perceived mental and physical health benefits. This means that many of the users had been using the app before being included in the sample; so, neither were the analyses able to characterize the initial behavior of app users nor could the results identify the initial period necessary to form a temporally similar meditation habit. In addition, the measure of meditation habits focused on the temporal dimensions of app use as no direct measure was collected on users’ behavioral context or environment. Future research that combines the temporal dynamics of daily behavior with additional contextual information could provide a more complete picture of this habit formation process. As temporal data are readily available from a wide range of health apps and other mHealth devices, this study provides a method for analyzing the habit formation process that can be readily applied across these different health behavior settings. Finally, not all reflexive behavioral habits will be performed at approximately the same time each day, and our measure of temporal similarity will only identify the habits that are initiated by temporally similar contextual cues. As past research has found that most habits are instigated by temporally similar contextual cues [25,26], a high degree of temporal similarity is an important indicator of reflexive habits but is not a necessary condition.

### Comparison With Prior Work

This study constructed a measure of temporally similar daily meditation app use based on the DTW distance between consecutive days, which adds to the bourgeoning literature using DTW distance to detect temporal patterns in a wide range of behavioral and health data settings [40-45]. One of the most common existing uses of DTW distance has been to categorize patterns of daily health behavior. For example, unhealthy dietary routines were identified by analyzing the variation in DTW distance between participants’ eating behaviors over 24-hour periods [43]. DTW distance measures have also been used to identify unhealthy sedentary behaviors [41] and to diagnose hyperactivity disorder from the patterns in children’s bodily movements while completing stationary computer tasks [42]. In a clinical setting, DTW-based measures have been used to identify early signs of kidney transplant rejection [40] and to categorize the experience level of surgeons [44]. Recent research has also used DTW-based measures for making predictions from longitudinal health data, such as predicting future glucose levels of patients with type 2 diabetes [45]. In this study, we extended this forecasting approach to the prediction of future health behaviors and showed that a DTW distance measure of temporal similarity in meditation app use on consecutive days significantly predicts an individual’s future meditation behavior. This study adds to the nascent literature that uses measures of temporal similarity to characterize health habits [46].

This paper also contributes to the mHealth literature characterizing mobile phone app engagement over time. Declining app use is an important concern and limitation of many app-based health promotion tools, and researchers have found that app engagement durations are becoming increasingly shorter [47]. This is consistent with declining engagement with other mHealth tools over the course of behavioral interventions, which has been observed for self-monitoring technologies, such as physical activity trackers [48] and glucose monitoring [49], as well as adherence to a telemonitoring program for heart failure [50]. This study shows that the temporal similarity of daily meditation app use is an important predictor of continued app use, which suggests that future app-based health promotional tools and interventions should also promote temporal similarity when providing health benefits that require consistent performance over time.

### Conclusions

Promoting healthier habits is an important public health objective for improving many health outcomes. However, to date, the study of habit formation has relied on self-reported measures and has yet to use the abundance of new behavioral health data being collected through mHealth devices. The field’s limited methods for measuring health habits and understanding of the habit formation process have likely contributed to the lack of behavioral health interventions that have successfully established persistent behavioral changes.

This study presents a novel objective indicator of reflexive habits derived from detailed, objective behavioral data collected by a mindfulness meditation mobile phone app. Our measure of temporal similarity in meditation app use on consecutive days significantly predicted future app use, even after controlling for users’ demographic and socioeconomic characteristics and common measures of app use, such as the frequency and duration of use. Importantly, this temporal similarity measure was also associated with greater odds of experiencing improved mental health from using the app, which suggests that forming a reflexive meditation habit may provide additional mental health benefits. This measure of temporal similarity can be readily applied to other sources of behavioral health data, and future research should build on these findings by investigating the ability of temporal similarity to identify habits in these other behavioral settings. In addition, future research should investigate whether reflexively initiated meditation habits can increase the mental health benefits from mindfulness meditation.

## Appendix

Multimedia Appendix 1 Description of the temporal similarity measure.

Multimedia Appendix 2 Visualizations outlining behavioral data from the mindfulness meditation app.

Multimedia Appendix 3 Random forest variable importance measures predicting future app use.

Multimedia Appendix 4 Logistic regressions of users’ future app use on demographic characteristics.

Multimedia Appendix 5 Predictive modeling results for alternative measures of users’ future app use.

## Abbreviations

AUC: area under the receiver operating characteristic curve
DTW: dynamic time warping
mHealth: mobile health
OR: odds ratio
ROC: receiver operating characteristic
