# Supplementary material for: Identifying App-Based Meditation Habits and the Associated Mental Health Benefits: Longitudinal Observational Study
Source: J Med Internet Res. 2021 Nov 4;23(11):e27282. doi: 10.2196/27282 (PMC8603170; doi:10.2196/27282)
Supplement: Multimedia Appendix 1 [file jmir_v23i11e27282_app1.docx]

**Appendix 1: Temporal similarity measure:**

Temporal similarity was estimated by converting each day in our sample period into a 1440-minute time series of zeros (when the app was not in use) and ones (when the app was being used), then calculating the DTW distance between the time series of app use on consecutive days. For these analyses, the time range in which the DTW algorithm was able to compare usage across days was restricted to 60 minutes. Figure S1 visualizes the DTW distance calculation with this time range restriction for a specific example in our data. In this example, app use of 20 minutes that was performed at 6:00pm on Day 1 and 7:00pm on Day 2 is considered equivalent by the DTW algorithm, while a second session of app use on Day 1 at 5:10pm results in a positive DTW distance. Specifically, the Euclidean distance between the two days is 50 minutes while the DTW distance is 10 minutes, which again highlights the flexibility of the DTW-based distance calculation. Importantly, this paper’s results were qualitatively unchanged when the window was expanded to 90 or 120 minutes. The DTW distance was estimated using the ‘dtw’ package in *R*, and the time range restriction was specified by setting the Sakoe-Chiba band equal to 60.

The measure of DTW distance between consecutive days was further modified to penalize users for consecutive days of no use in order to better capture temporally consistent app usage. Since the DTW distance between consecutive days of non-use is zero, the zero DTW measure on all days of consecutive non-use (and all subsequent, consecutive days of non-use) was replaced with the maximum DTW distance observed over the 14-day interval that included the given day of non-use. Under this construction, the penalized version of the DTW measure will only decline when users’ actual app usage becomes more similar, and not simply when they stop using the app entirely. In robustness exercises, the non-use penalty was also set to the median DTW distance observed within the corresponding 14-day interval, and the results were both quantitatively and qualitatively similar. Finally, the penalized DTW measure was standardized across users by dividing each daily DTW measure by users’ average duration (in minutes) of daily app use over the corresponding 14-day interval. This way, frequent users with a large average number of daily minutes who would generate a larger DTW distance measure for skipping a day, or otherwise reducing their usage, are more readily comparable with less frequent users. The final DTW measure can be interpreted as the average percent of daily minutes that are inconsistently performed between consecutive days over each 14-day interval.

**Observed behavioral data:**

Figures S2 and S3 display the range of app data that inform the objective measures of app use, and illustrate the relationship between the DTW-based measure of temporal similarity and users’ future app use. The first panel in each figure shows the total duration of daily app use in minutes, the second panel shows the start time of each daily session, and the last panel shows the daily session count by session type. Additionally, the DTW distance measure calculated over the first 10 14-day intervals for each user is included in first panel. In Figure S2, this user consistently uses a single meditation session around midnight (minute 1,439; 11:59pm) over the first 10 14-day intervals, which is reflected in the declining DTW time series over the same period. Importantly, this pattern of meditation in the evenings continued uninterrupted for the remaining days in our sample. Conversely, in Figure S3, the user varied their use of both meditation and sleep stories in the evenings (minutes 0-60; 12am-1am), mornings (minute 510; 8:30 am), and later throughout the day during the first 10 14-day intervals, which caused the DTW time series to steadily increase over this period. Over the subsequent days in our sample, the user in Figure S3 displayed inconsistent app usage with a declining frequency. The study evaluated the statistical significance of this relationship between DTW distance and subsequent app usage over the full sample, as well as the association between DTW distance and users’ perceived mental benefits from using the app.
