# Supplementary material for: Identifying App-Based Meditation Habits and the Associated Mental Health Benefits: Longitudinal Observational Study
Source: J Med Internet Res. 2021 Nov 4;23(11):e27282. doi: 10.2196/27282 (PMC8603170; doi:10.2196/27282)
Supplement: Multimedia Appendix 2 [file jmir_v23i11e27282_app2.docx]

**
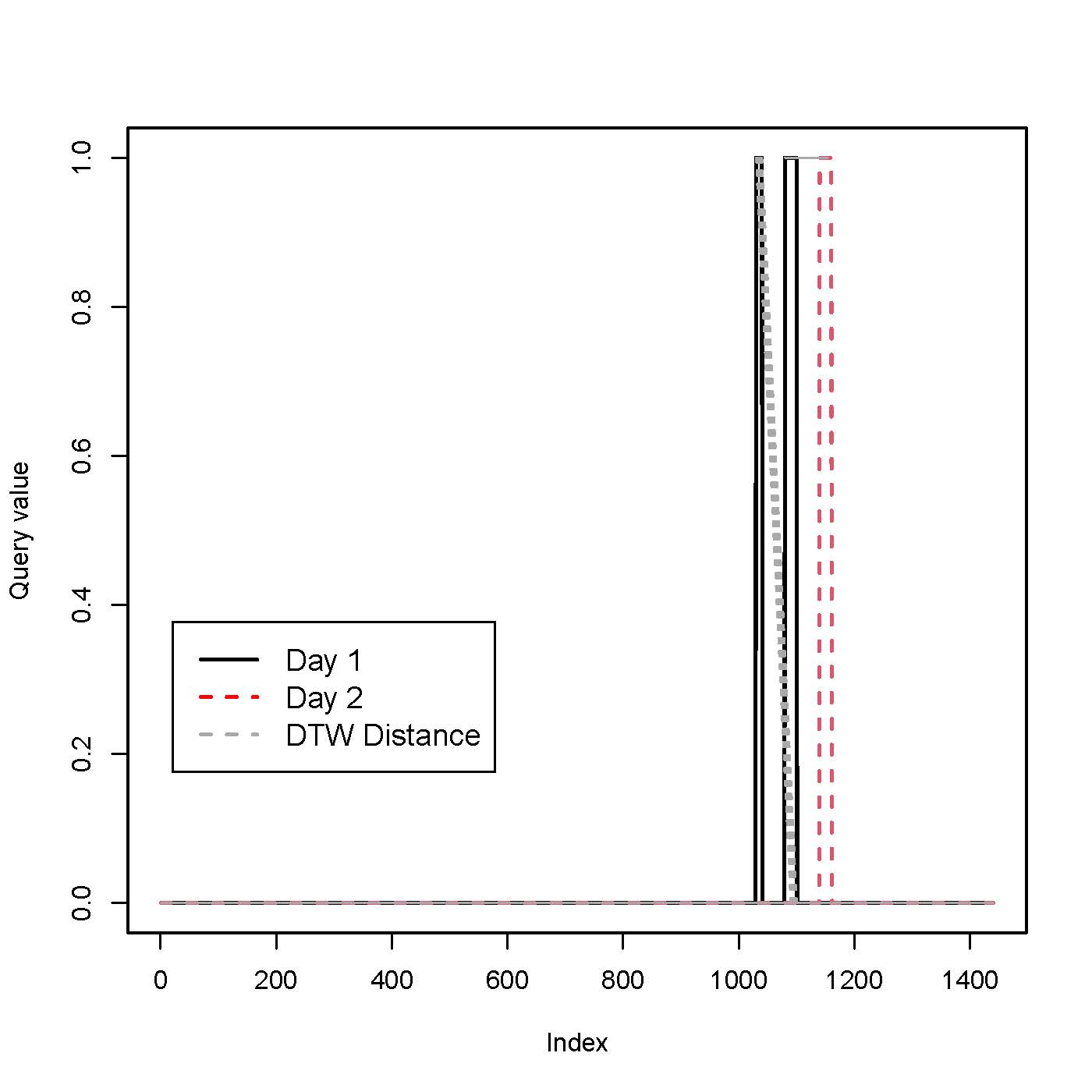
**

**Figure S1**: A visualization of the DTW distance between two consecutive days of use for single app user in our data. The grey dotted lines illustrate which values are compared, where usage (indicated by a one) is plotted by the minute of the day (i.e. 0 = 12:10am and 1,439 = 1:59pm). The DTW distance is calculated using a restricted window of 60 minutes, so app use of equal length performed within +/- 60 minutes of each other on Day 1 and Day 2 are compared using the DTW distance. In this example, 20 minutes of meditation was performed at 6:00pm on Day 1 and 7pm on Day 2, so the DTW distance between these sessions is 0. The additional 10 minutes app use on Day 1 that was performed at 5:10pm, more than 60 minutes before use on Day 2, is compared to periods of non-use on Day 2, and thus the total DTW distance between these two days is equal to 10. The Euclidean distance between these two days is 50.

**
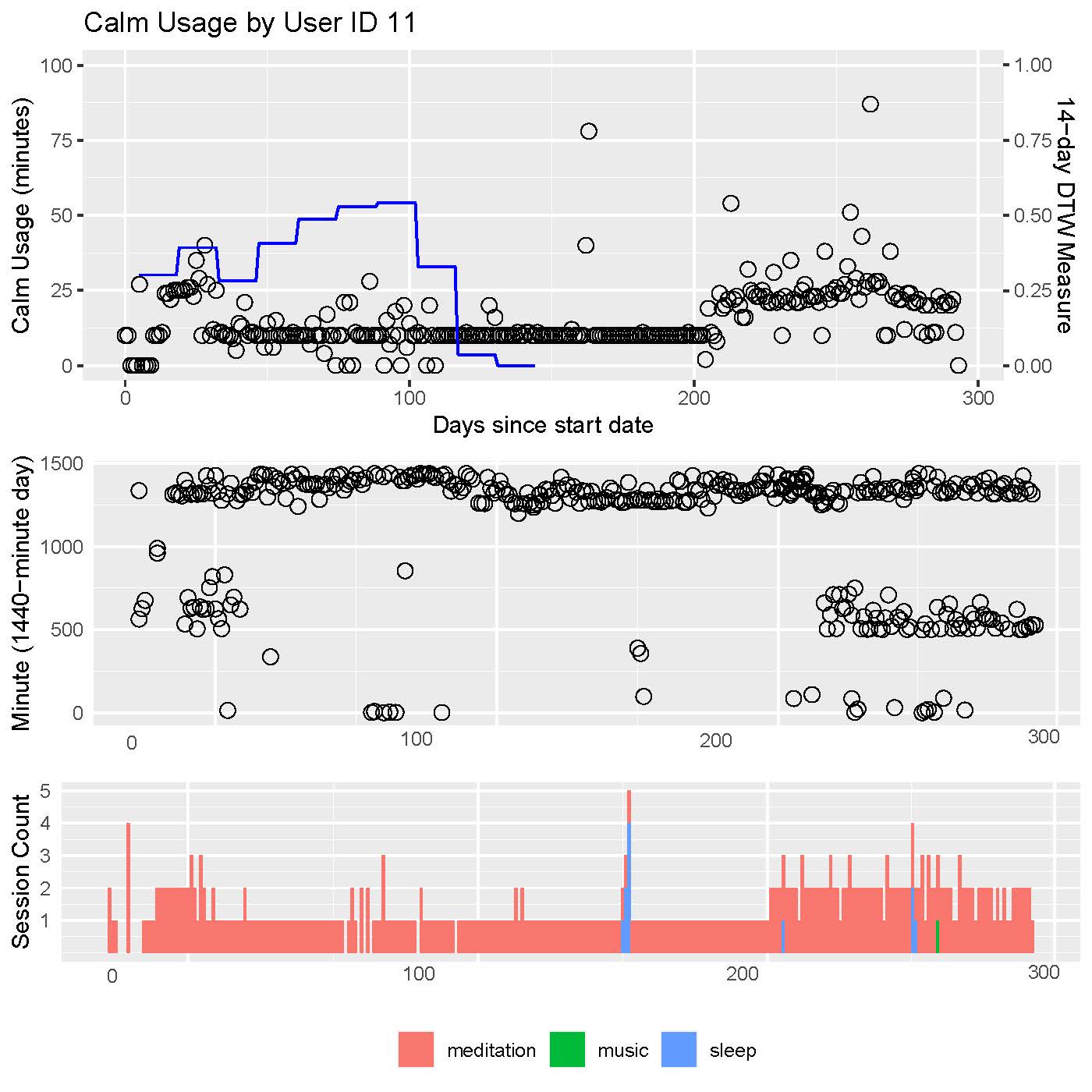
**

**Figure S2:** This figure displays the primary data features that were used to construct objective measures of app use. The top panel displays the total duration of daily usage in minutes, the second panel shows the start time of each unique daily session, and the final panel shows a daily count of sessions by type. The top panel additionally displays a time series of the DTW distance calculation for the first 10 14-day intervals from the user’s start date. This user displays strong temporal consistency in daily meditation sessions which results in a decreasing DTW distance over time, and they are persistent in their daily meditation over the full sample period.

**
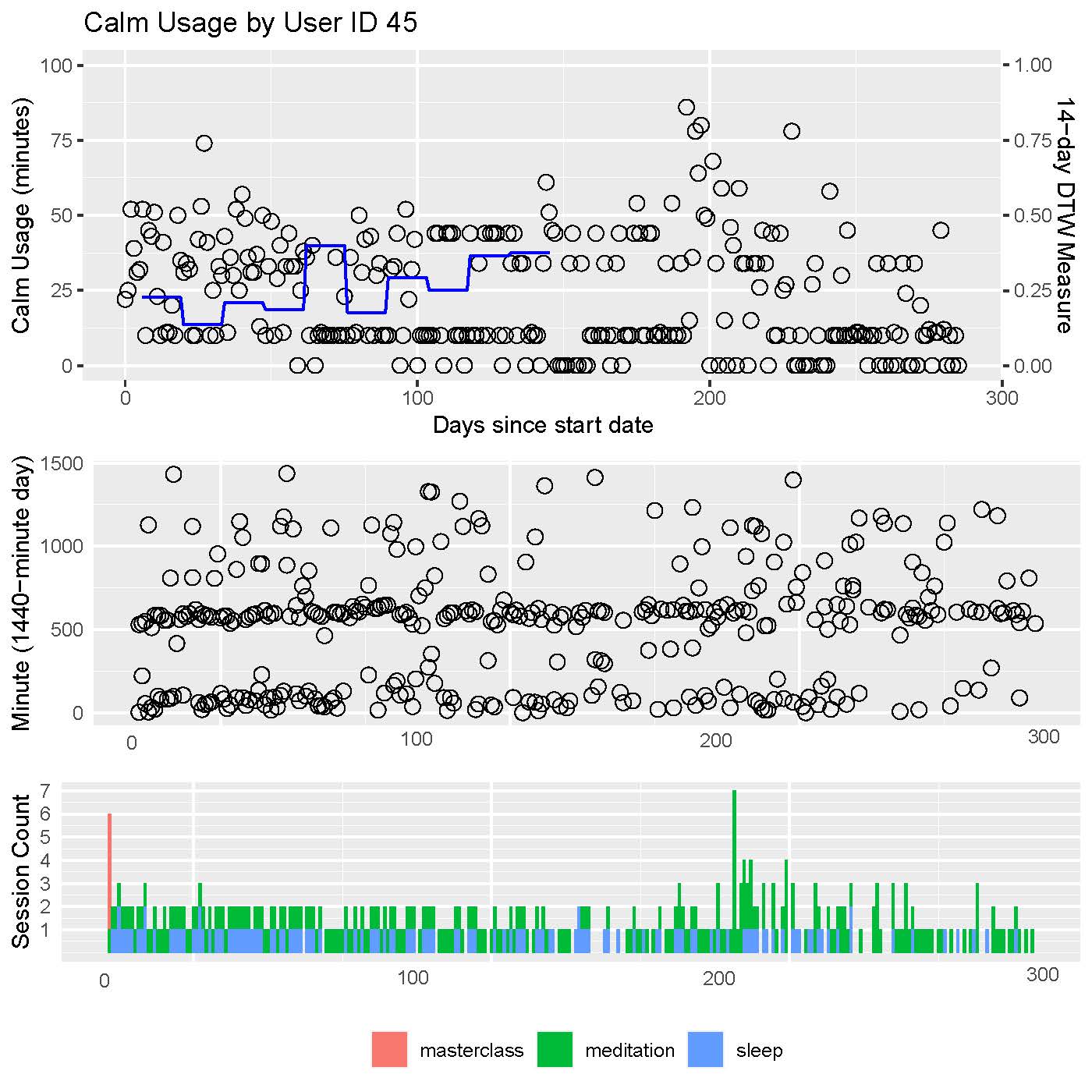
**

**Figure S3:** This figure displays the primary data features that were used to construct objective measures of app use. The top panel displays the total duration of daily usage in minutes, the second panel shows the start time of each unique daily session, and the final panel shows a daily count of sessions by type. The top panel additionally displays a time series of the DTW distance calculation for the first 10 14-day intervals from the user’s start date. This user varies their daily timing of app use which results in an increasing DTW distance over time, and they become less consistent in their app use towards the end of the sample period.
