# Supplementary material for: Identifying App-Based Meditation Habits and the Associated Mental Health Benefits: Longitudinal Observational Study
Source: J Med Internet Res. 2021 Nov 4;23(11):e27282. doi: 10.2196/27282 (PMC8603170; doi:10.2196/27282)
Supplement: Multimedia Appendix 3 [file jmir_v23i11e27282_app3.docx]

**Table S1: Variable importance from random forests predicting future app use**

|  | Any Use 28 Days Later | Any Use 28 Days Later | Any Use 6 Months Later | Any Use 6 Months Later | High Duration in Next 28 Days | High Duration in Next 28 Days |
| --- | --- | --- | --- | --- | --- | --- |
| Days of any use: Interval 10 | 1.000 | 0.862 | 0.732 | 0.615 | 0.086 | 0.094 |
| Days of any use: Interval 9 | 0.862 | 0.789 | 0.406 | 0.502 | 0.191 | 0.098 |
| Days of any use: Interval 8 | 0.560 | 0.310 | 0.324 | 0.273 | 0.091 | 0.084 |
| Days of any use: Interval 7 | 0.275 | 0.249 | 0.469 | 0.320 | 0.059 | 0.063 |
| Days of any use: Interval 6 | 0.304 | 0.174 | 0.492 | 0.387 | 0.050 | 0.079 |
| Total sessions: Interval 10 | 0.810 | 1.000 | 1.000 | 1.000 | 0.180 | 0.110 |
| Total sessions: Interval 9 | 0.952 | 0.561 | 0.741 | 0.498 | 0.165 | 0.094 |
| Total sessions: Interval 8 | 0.441 | 0.335 | 0.369 | 0.389 | 0.156 | 0.075 |
| Total sessions: Interval 7 | 0.467 | 0.381 | 0.631 | 0.463 | 0.076 | 0.079 |
| Total sessions: Interval 6 | 0.130 | 0.093 | 0.511 | 0.394 | 0.093 | 0.106 |
| Total duration: Interval 10 | 0.174 | 0.125 | 0.574 | 0.447 | 1.000 | 1.000 |
| Total duration: Interval 9 | 0.257 | 0.155 | 0.535 | 0.464 | 0.974 | 0.764 |
| Total duration: Interval 8 | 0.351 | 0.202 | 0.507 | 0.339 | 0.169 | 0.143 |
| Total duration: Interval 7 | 0.220 | 0.247 | 0.511 | 0.542 | 0.187 | 0.171 |
| Total duration: Interval 6 | 0.217 | 0.181 | 0.533 | 0.402 | 0.263 | 0.205 |
| DTW average: Interval 10 |  | 0.453 |  | 0.546 |  | 0.416 |
| DTW average: Interval 9 |  | 0.197 |  | 0.406 |  | 0.286 |
| DTW average: Interval 8 |  | 0.141 |  | 0.359 |  | 0.208 |
| DTW average: Interval 7 |  | 0.145 |  | 0.392 |  | 0.542 |
| DTW average: Interval 6 |  | 0.112 |  | 0.374 |  | 0.591 |

**Note**: Each column presents the variable importance measures from random forest models predicting the outcome indicated by the column title using the predictors indicated by row titles; intervals 5 – 1 were also included in the model but are suppressed from the table. The top five variable importance statistics are highlighted in grey, and all importance measures were divided by the maximum variable importance statistic in each model.
