# Supplementary material for: Identifying App-Based Meditation Habits and the Associated Mental Health Benefits: Longitudinal Observational Study
Source: J Med Internet Res. 2021 Nov 4;23(11):e27282. doi: 10.2196/27282 (PMC8603170; doi:10.2196/27282)
Supplement: Multimedia Appendix 4 [file jmir_v23i11e27282_app4.docx]

**Table S1: Future app usage on demographics**

|  |  |  |  |
| --- | --- | --- | --- |
|  | Any Use 28 Days Later | Any Use 6 Months Later | High Duration in Next 28 Days |
|  |  |  |  |
| 31 - 40 y.o. | 1.244 | 1.138 | 1.260 |
|  | [0.939,1.646] | [0.832,1.556] | [0.951,1.668] |
| 41 - 50 y.o. | 1.389^a^ | 1.164 | 1.420^a^ |
|  | [1.047,1.842] | [0.851,1.592] | [1.070,1.884] |
| 51 - 60 y.o. | 1.428^a^ | 1.429^a^ | 1.314 |
|  | [1.071,1.903] | [1.033,1.975] | [0.986,1.750] |
| 61 - 70 y.o. | 2.165^c^ | 2.012^c^ | 2.040^c^ |
|  | [1.585,2.957] | [1.406,2.881] | [1.496,2.781] |
| 71 - 80 y.o. | 2.740^c^ | 2.593^c^ | 2.834^c^ |
|  | [1.745,4.304] | [1.503,4.473] | [1.796,4.473] |
| 81+ y.o. | 1.225 | 0.907 | 1.611^a^ |
|  | [0.778,1.929] | [0.556,1.479] | [1.020,2.543] |
| Female | 0.913 | 1.043 | 1.159 |
|  | [0.727,1.147] | [0.804,1.354] | [0.926,1.450] |
| White | 1.362^a^ | 1.253 | 1.050 |
|  | [1.002,1.851] | [0.884,1.774] | [0.773,1.425] |
| Asian | 0.579 | 0.751 | 0.884 |
|  | [0.332,1.011] | [0.422,1.338] | [0.518,1.509] |
| Black | 1.138 | 0.836 | 0.895 |
|  | [0.655,1.978] | [0.454,1.537] | [0.516,1.553] |
| Hispanic | 0.731 | 0.969 | 0.653^a^ |
|  | [0.514,1.040] | [0.650,1.445] | [0.459,0.927] |
| Log(Income) | 1.042 | 1.096 | 0.952 |
|  | [0.945,1.150] | [0.994,1.209] | [0.864,1.048] |
| Employed full-time | 1.144 | 1.333^b^ | 0.954 |
|  | [0.955,1.370] | [1.086,1.635] | [0.797,1.142] |
| Bachelor’s degree | 0.955 | 1.150 | 0.813^a^ |
|  | [0.780,1.170] | [0.911,1.453] | [0.664,0.995] |
| Graduate degree | 0.891 | 1.038 | 0.866 |
|  | [0.725,1.095] | [0.819,1.315] | [0.705,1.064] |
| **Observations** | **2,771** | **2,771** | **2,771** |

Odds ratios (exponentiated coefficients); 95% confidence intervals in brackets.

^a^ *P* < 0.05, ^b^ *P* < 0.01, ^c^ *P* < 0.001
