# Supplementary material for: Identifying App-Based Meditation Habits and the Associated Mental Health Benefits: Longitudinal Observational Study
Source: J Med Internet Res. 2021 Nov 4;23(11):e27282. doi: 10.2196/27282 (PMC8603170; doi:10.2196/27282)
Supplement: Multimedia Appendix 5 [file jmir_v23i11e27282_app5.docx]

**Table S1: Objective measures predicting future app usage over the next 28 days**

|  | (1) | (2) | (3) | (4) |
| --- | --- | --- | --- | --- |
|  | Above Median Meditation Sessions | Above Median Meditation Sessions | Above Median Sleep Stories | Above Median Sleep Stories |
| Log(Days of any use): Int. 10 | 1.787^c^ | 1.722^a^ | 1.599^b^ | 1.457 |
|  | [1.27,2.51] | [1.06,2.79] | [1.20,2.13] | [0.87,2.44] |
| Log(Days of any use): Int. 9 | 2.023^c^ | 1.595 | 2.132^c^ | 2.034^b^ |
|  | [1.38,2.96] | [0.95,2.67] | [1.56,2.92] | [1.24,3.34] |
| Log(Days of any use): Int. 8 | 1.808^a^ | 1.632 | 0.860 | 0.465^a^ |
|  | [1.15,2.85] | [0.93,2.86] | [0.59,1.26] | [0.26,0.84] |
| Log(Days of any use): Int. 7 | 1.228 | 1.330 | 1.101 | 0.640 |
|  | [0.79,1.90] | [0.79,2.24] | [0.75,1.63] | [0.38,1.09] |
| Log(Days of any use): Int. 6 | 0.959 | 0.870 | 0.953 | 0.617 |
|  | [0.63,1.46] | [0.52,1.45] | [0.65,1.39] | [0.37,1.03] |
| Log(Total sessions): Int. 10 | 1.632 | 1.681 | 1.395 | 1.165 |
|  | [0.99,2.70] | [0.99,2.84] | [0.83,2.34] | [0.66,2.07] |
| Log(Total sessions): Int. 9 | 1.902^a^ | 1.804 | 0.606 | 0.473^b^ |
|  | [1.07,3.37] | [0.98,3.32] | [0.37,1.00] | [0.27,0.83] |
| Log(Total sessions): Int. 8 | 0.946 | 1.050 | 1.482 | 1.119 |
|  | [0.54,1.65] | [0.59,1.88] | [0.88,2.49] | [0.61,2.05] |
| Log(Total sessions): Int. 7 | 1.890^a^ | 1.865^a^ | 1.109 | 1.161 |
|  | [1.13,3.16] | [1.09,3.19] | [0.64,1.91] | [0.63,2.13] |
| Log(Total sessions): Int. 6 | 1.091 | 1.087 | 0.981 | 0.998 |
|  | [0.64,1.86] | [0.63,1.86] | [0.57,1.70] | [0.56,1.77] |
| Log(Total duration): Int. 10 | 0.791^c^ | 0.856^b^ | 0.864^c^ | 0.776^c^ |
|  | [0.72,0.86] | [0.77,0.96] | [0.80,0.94] | [0.69,0.87] |
| Log(Total duration): Int. 9 | 0.715^c^ | 0.747^c^ | 0.777^c^ | 0.846^a^ |
|  | [0.65,0.79] | [0.66,0.84] | [0.71,0.85] | [0.74,0.97] |
| Log(Total duration): Int. 8 | 0.761^c^ | 0.733^c^ | 0.996 | 1.203^a^ |
|  | [0.67,0.87] | [0.64,0.84] | [0.89,1.11] | [1.01,1.44] |
| Log(Total duration): Int. 7 | 0.834^b^ | 0.799^b^ | 0.909 | 0.951 |
|  | [0.73,0.95] | [0.70,0.91] | [0.81,1.02] | [0.81,1.11] |
| Log(Total duration): Int. 6 | 0.901 | 0.932 | 0.985 | 1.048 |
|  | [0.79,1.02] | [0.82,1.06] | [0.88,1.10] | [0.90,1.22] |
| Log(DTW distance): Int. 10 |  | 0.326^b^ |  | 0.0484^c^ |
|  |  | [0.11,0.76] |  | [0.01,0.25] |
| Log(DTW distance): Int. 9 |  | 0.318^a^ |  | 0.237^a^ |
|  |  | [0.12,0.67] |  | [0.06,0.88] |
| Log(DTW distance): Int. 8 |  | 0.685 |  | 0.165^a^ |
|  |  | [0.24,2.00] |  | [0.04,0.66] |
| Log(DTW distance): Int. 7 |  | 0.731^a^ |  | 0.219^a^ |
|  |  | [0.33,0.84] |  | [0.06,0.79] |
| Log(DTW distance): Int. 6 |  | 0.708 |  | 0.336^a^ |
|  |  | [0.25,2.02] |  | [0.12,0.94] |
| All usage measures: Int. 5 - 1 | x | x | x | x |
| Demographic controls | x | x | x | x |
| **Observations** | **2,771** | **2,771** | **2,771** | **2,771** |

Odds ratios (exponentiated coefficients); 95% confidence intervals in brackets; ^a^ *P* < 0.05, ^b^ *P* < 0.01, ^c^ *P* < 0.001

| **Table S2: Area under the ROC curve without and with DTW to predict future app usage** | | | | | | | |
| --- | --- | --- | --- | --- | --- | --- | --- |
|  | Above Median Meditation Sessions Over Next 28 days | | | Above Median Sleep Story Sessions Over Next 28 days | | |  |
| **Predictors calculated:** | | |  | | |  | |
| By aggregating all sessions: | | |  | | |  | |
| Not including DTW | | 0.779 | | | 0.804 | |  |
| Including DTW | | 0.783 | | | 0.805 | |  |
| **Difference (*p-value*)** | | 0.004 (0.007) | | | 0.001 (0.198) | |  |
| By session timing (weekday, weekend): | | |  | | |  | |
| Not including DTW | | 0.795 | | | 0.813 | |  |
| Including DTW | | 0.839 | | | 0.849 | |  |
| **Difference (*p-value*)** | | 0.044 (<0.001) | | | 0.036 (<0.001) | |  |
| By session type and timing: | | |  | | |  | |
| Not including DTW | | 0.946 | | | 0.942 | |  |
| Including DTW | | 0.952 | | | 0.952 | |  |
| **Difference (*p-value*)** | | 0.006 (0.003) | | | 0.010 (<0.001) | |  |
